# Food Insufficiency Is Associated with High-Risk Sexual Behavior among Women in Botswana and Swaziland

**DOI:** 10.1371/journal.pmed.0040260

**Published:** 2007-10-23

**Authors:** Sheri D Weiser, Karen Leiter, David R Bangsberg, Lisa M Butler, Fiona Percy-de Korte, Zakhe Hlanze, Nthabiseng Phaladze, Vincent Iacopino, Michele Heisler

**Affiliations:** 1 Physicians for Human Rights, Cambridge, Massachusetts, United States of America; 2 Epidemiology and Prevention Interventions Center, San Francisco General Hospital, University of California San Francisco, San Francisco, California, United States of America; 3 Center for AIDS Prevention Studies, University of California San Francisco, San Francisco, California, United States of America; 4 Positive Health Program, San Francisco General Hospital, University of California San Francisco, San Francisco, California, United States of America; 5 Department of Epidemiology and Biostatistics, University of California San Francisco, San Francisco, California, United States of America; 6 Women and Law in Southern Africa Research Trust, Mbabane, Swaziland; 7 Department of Nursing, University of Botswana, Gaborone, Botswana; 8 Department of Medicine, University of Minnesota, Minneapolis, Minnesota, United States of America; 9 Department of Internal Medicine, University of Michigan School of Medicine, Ann Arbor, Michigan, United States of America; 10 VA Center for Practice Management and Outcomes Research, Ann Arbor Veterans Affairs Health System, Ann Arbor, Michigan, United States of America; International Food Policy Research Institute, United States of America

## Abstract

**Background:**

Both food insufficiency and HIV infection are major public health problems in sub-Saharan Africa, yet the impact of food insufficiency on HIV risk behavior has not been systematically investigated. We tested the hypothesis that food insufficiency is associated with HIV transmission behavior.

**Methods and Findings:**

We studied the association between food insufficiency (not having enough food to eat over the previous 12 months) and inconsistent condom use, sex exchange, and other measures of risky sex in a cross-sectional population-based study of 1,255 adults in Botswana and 796 adults in Swaziland using a stratified two-stage probability design. Associations were examined using multivariable logistic regression analyses, clustered by country and stratified by gender. Food insufficiency was reported by 32% of women and 22% of men over the previous 12 months. Among 1,050 women in both countries, after controlling for respondent characteristics including income and education, HIV knowledge, and alcohol use, food insufficiency was associated with inconsistent condom use with a nonprimary partner (adjusted odds ratio [AOR] 1.73, 95% confidence interval [CI] 1.27–2.36), sex exchange (AOR 1.84, 95% CI 1.74–1.93), intergenerational sexual relationships (AOR 1.46, 95% CI 1.03–2.08), and lack of control in sexual relationships (AOR 1.68, 95% CI 1.24–2.28). Associations between food insufficiency and risky sex were much attenuated among men.

**Conclusions:**

Food insufficiency is an important risk factor for increased sexual risk-taking among women in Botswana and Swaziland. Targeted food assistance and income generation programs in conjunction with efforts to enhance women's legal and social rights may play an important role in decreasing HIV transmission risk for women.

## Abstract 2

In a cross-sectional study, Sheri Weiser and colleagues found that food insufficiency was an important risk factor for increased sexual risk-taking in women in Botswana and Swaziland.

## Editors' Summary

**Background.:**

For people in sub-Saharan Africa, insufficient food for their daily needs and infection with the human immunodeficiency virus (HIV; the cause of acquired immunodeficiency syndrome or AIDS) are inextricably linked and major causes of illness and death. By reducing the number of healthy adults in the region, HIV/AIDS has decreased food production so fewer people have secure access to sufficient food for a healthy life—many are subject to “food insecurity.” Because good nutrition is essential for a strong immune system, food insecurity increases the likelihood that people exposed to HIV become infected with the virus and reduces their ability to remain healthy after infection. Consequently, more people succumb to HIV/AIDS and food insecurity increases. To break this vicious cycle, UNAIDS (the Joint United Nations Programme on HIV/AIDS) and other international bodies have suggested a move towards integrated food security programs and HIV/AIDS prevention and treatment programs wherever possible.

**Why Was This Study Done?:**

Integrated food and HIV/AIDS programs might also be beneficial for another reason. HIV is usually spread through unprotected sex with an infected partner and it is thought that a lack of food increases sexual risk taking, particularly among poor women. These women have little control over food supplies but are expected to feed their children and other members of the household (such as elders). To do this, they may sell sex or become sexually involved with men of a different generation, both of which put them at risk of HIV. In addition, they are rarely able to demand that their partners use condoms or to control when they have sex. In this study, the researchers have examined how food insufficiency affects sexual risk taking among men and women in Swaziland and Botswana. These two countries have the highest HIV infection rates in the world—one in three adults in Swaziland and one in four in Botswana are infected—and in both countries many people are extremely poor.

**What Did the Researchers Do and Find?:**

The researchers interviewed more than 2,000 randomly selected adults from Botswana and Swaziland using a standard questionnaire. This included general questions about the participants (for example, age and marital status) and questions about food insufficiency (defined as not having had enough food to eat over the previous 12 months) and risky sexual behaviors—for example, sex exchange (selling or paying for sex) and inconsistent condom use—over the same period. Nearly one in three women and one in four men reported food insufficiency. After allowing for variables such as education and income, women in both countries who reported food insufficiency were nearly twice as likely to have used condoms inconsistently with a non-regular partner or to have sold sex as women who had had sufficient food. They were also more likely to have had intergenerational sexual relationships and to report a lack of control in sexual relationships. Among men, food insufficiency was weakly associated with inconsistent condom use but not with other risky sexual behaviors.

**What Do These Findings Mean?:**

Food insufficiency is associated with multiple (often interdependent) risky sexual practices among women in Botswana and Swaziland. These results may not hold for other countries and may be limited by the definition of food insufficiency used in the study and by participants failing to remember or report all instances of risky behavior or food insufficiency that occurred during the previous year. Nevertheless, the findings strongly suggest that protecting and promoting access to food may decrease vulnerability of women in sub-Saharan Africa to HIV infection. Improved food security might be achieved through targeted food assistance and by supporting women's subsistence farming and other means of food production. Such programs would also need to enhance women's legal and social rights so that they have more control over food supplies as well as their sexual lives.

**Additional Information.:**

Please access these Web sites via the online version of this summary at http://dx.doi.org/10.1371/journal.pmed.0040260.

- The Food and Agriculture Organization of the United Nations Special Programme for Food Security (in several languages)
- HIV InSite, comprehensive and up-to-date information on all aspects of HIV/AIDS from the University of California San Francisco
- UNAIDS (Joint United Nations Programme on HIV/AIDS) fact sheet on nutrition, food security, and HIV/AIDS
- HIV and AIDS in Swaziland and Botswana, information provided by Avert, an international AIDS charity
- The IMAGE Study, an example of a research initiative that is investigating the potential role of poverty alleviation and women's empowerment in reducing HIV incidence in South Africa

## Introduction

HIV and food insufficiency (lacking adequate food supply to meet daily needs) are two leading causes of morbidity and mortality in sub-Saharan Africa and are thought to be inextricably linked [1,2]. In addition to the well-documented negative impacts of HIV/AIDS on food security in Africa [1,3–5], there is a growing recognition by international health organizations and nongovernmental organizations that food insufficiency might increase HIV transmission risk behaviors and susceptibility to HIV once exposed [1,6]. Accordingly, the World Health Organization, UNAIDS, the World Food Program, and other international organizations have suggested a move toward integrating food and HIV/AIDS programming activities where possible [6–9].

To date, however, there have been few studies of the effect of food insufficiency on HIV transmission. Stillwaggon [10] found that the prevalence of HIV in 44 sub-Saharan African settings was highly correlated with declining energy and protein consumption. Similarly, Moore et al. [11] reported that weight loss in Rwanda was associated with eventual HIV seroconversion among women. Yet the mechanisms by which food insufficiency may lead to increased HIV transmission and acquisition have not been adequately characterized. One postulated mechanism is that lack of food and malnutrition weaken the immune system and can compromise gut and genital mucosal integrity, thereby increasing vulnerability to HIV once exposed [1,12]. Food insecurity is also hypothesized to increase sexual risk-taking—especially among women living in poverty who are often dependent on others for food and other resources, and whose human rights are inadequately protected [13]. Women in parts of sub-Saharan Africa such as Botswana and Swaziland often lack control over resources, including the food supply at home, while also bearing responsibility for caring for children, elder household members, and household members who are ill [1,14–16]. Previous qualitative research has shown that women may engage in sex exchange or get involved with intergenerational relationships in order to procure food for themselves and their children [1,8,17,18]. In a cross-sectional study by Oyefara et al. among 320 female sex workers in Lagos, Nigeria, 35% of respondents said that poverty and lack of other means to get food was responsible for their decision to become sex workers [19]. In addition, as a result of dependence on their partner for food and other resources, women may have little control over condom use [17]. For example, qualitative studies in Botswana and Swaziland have found that women often lack power to negotiate for safer sex in their relationships, a situation exacerbated by their poor socioeconomic circumstances [13,16,20]. While few studies have examined the specific impact of food insufficiency on sexual risk-taking, a number of studies have shown that low socioeconomic status is associated with sex exchange, coerced sex, and inconsistent condom use in African countries [1,21–25]. Conversely, greater negotiating power within sexual relationships and economic independence were found to be positively associated with condom use in a small cross-sectional study among 71 women in Gaborone, Botswana [26].

To our knowledge, no studies have quantitatively examined the independent impact of food insufficiency on sexual risk-taking, and there are no data on this critical issue from population-based studies. In addition, there are no data on how gender modifies the association between food insufficiency and risky sexual behavior, or whether the effects of food insufficiency on sexual risk are mediated primarily by income. To address these gaps, this study had two aims: (1) To assess the association between food insufficiency and risky sexual behaviors while controlling for other key respondent characteristics, including income and education. The risky sexual behaviors that we examined include inconsistent condom use with a nonprimary partner, sex exchange (selling or paying for sex in exchange for money or resources), intergenerational sexual relationships (having had sexual relations with a partner 10 or more years older or younger), lack of control in sexual relationships (the participant's partner usually or always made the decision as to whether or not to have sex), and forced sex; and (2) to determine whether gender modifies these associations.

## Methods

### Population and Setting

We conducted a population-based cross-sectional study between November 2004 and May 2005 among adults randomly selected from households in the five districts of Botswana with the highest number of HIV-infected individuals (Gaborone, Kweneng East, Francistown, Serowe/Palapye, and Tutume) and in all four districts of Swaziland. We used a stratified two-stage probability sample design for the selection of the population-based sample with the assistance of local experts from the Central Statistics Office in each country. First, 89 enumeration areas were selected in Botswana and 54 enumeration areas were selected in Swaziland with probability proportional to measures of size. At the second stage of sampling, households were systematically selected in the field from a list of occupied households prepared prior to the onset of the field work. Field supervisors monitored research assistants to ensure that households were appropriately selected and performed random checks on household selection as an additional quality control measure. Within each household, researchers used random number tables to select one adult member that met our inclusion criteria. Up to two repeat visits were made with no participant substitutions in the event that the selected participant could not be reached.

All research assistants were country nationals, had previous research experience, and received a 3- to 5-day training session on informed consent procedures and survey instruments prior to implementation. Our 45–60 min structured survey instruments were adapted to the local context through key informant interviews, and then pilot-tested on 20 individuals in Botswana and 29 individuals in Swaziland. All surveys and consent forms were translated into the local language (either Setswana or siSwati) and then back-translated into English to ensure that translations were accurate. Participants were included in the study if they were between the ages of 18 and 49 years, had no cognitive disabilities, were residents of the country where the interview took place, were fluent in English or siSwati or Setswana (the most common local languages), and if the interview could be conducted in reasonable privacy.

### Informed Consent

Written consent was obtained from all participants in Botswana according to local Institutional Review Board regulations. Oral consent was obtained in Swaziland according to WHO guidelines [27], as it was thought to be more culturally appropriate by in-country investigators due to high rates of illiteracy. In Botswana, study procedures were approved by the Human Subjects Committee at University of California, San Francisco, as well as by the Botswana Ministry of Health Research and Development Committee. In Swaziland, study procedures were approved by an Ethics Review Board convened by Physicians for Human Rights based in Boston, which included key stakeholders in Swaziland from the government, the University of Swaziland, and local nongovernmental organizations. The Swaziland research protocol and instruments were also approved by the chair of the newly reconstituted Ethics Committee of the Swaziland Ministry of Health. In both countries, all participants were offered information on HIV transmission and prevention, on domestic violence support groups, and on reporting domestic violence and rape to the police according to national law. If any participant appeared to be in emotional distress after answering sensitive questions, they were offered the opportunity to speak to one of the study health care providers and were referred to a local health care center for counseling.

### Measurements

Domains of inquiry for our survey included demographics, food insufficiency, HIV knowledge, measures of health care access and utilization, HIV risk behaviors, and beliefs about gender roles and gender discrimination. Food insufficiency, our primary independent variable, was defined as reporting not having enough food to eat over the previous 12 mo, a question modified from a previously validated measure of food insufficiency, which also used a single question [28]. To measure HIV prevention knowledge, questions were modified from the UNAIDS General Population Survey and the US Department of Health Services AIDS module. Using the UNAIDS knowledge indicator scoring system, individuals were scored as having HIV knowledge if they correctly identified the two most common modes of HIV prevention in each respective country (using condoms consistently and abstinence) [29]. To measure alcohol use, we asked participants to indicate the number of days per week that they drank alcohol, as well as the number of drinks per day on the days that they drank. Alcohol use was defined in three categories: none or moderate drinking; problem drinking, defined as 8–14 drinks/wk for women, 15–21 drinks/wk for men, which corresponds to the definition of risky drinking according to the National Institute of Alcohol Abuse and Alcoholism [30]; and heavy drinking, defined as more than 14 drinks/wk for women and 21 drinks/wk for men.

### Statistical Analysis

Data were analyzed with Stata statistical software version 8.0. We used multivariate logistic regression to assess the association between food insufficiency and a number of sexual behaviours or experiences that put individuals at risk for HIV. Key outcomes included: (1) inconsistent condom use with a nonprimary partner over the previous month; (2) sex exchange (for women this was defined as exchanging sex for money, food, or other resources over the previous 12 mo, and for men this was defined as paying for or providing resources for sex over the previous 12 mo); (3) intergenerational relationships, defined as having had sexual relations with a partner 10 or more years older or younger in the previous 12 mo [31] (this variable was included based on our previous findings that intergenerational sex is a strong marker of HIV risk behavior, including unprotected sex with a nonprimary partner and sex exchange [32], as well as our qualitative data suggesting that lack of food drives women to enter into these type of relationships); (4) lack of control in sexual decision-making in the previous 12 mo (participants were included in this category if they believed that their partner usually or always made the decision as to whether or not to have sex); and (5) having experienced forced sex over the previous 12 mo (exact phrasing: “Were you forced to have sex against your will over the past 12 months?”). Lack of control in sexual decision-making and having experienced forced sex were examined for women only since very few men reported either of these outcomes.

To account for the likely heterogeneity of responses between countries, and the fact that participants within each country were more likely to be similar to one another than to respondents in the other country, we adjusted the standard errors in all multivariate logistic regression models using the Huber/White heteroscedastic consistent estimator of the variance/covariance matrix with cluster correction, as found in Stata version 8 [33,34]. We stratified all analyses by gender, because we hypothesized that gender may differentially influence the association between food insufficiency and sexual practices. Covariates included in all multivariate analyses were: (1) age (continuous +1 y); (2) education (≥ high school, < high school); (3) residence type (rural, urban including small urban villages); (4) marital status (married, living with partner, other); (5) annual household income (dichotomized at the ordinal variable closest to the sample median in each country); (6) knowledge about HIV/AIDS; and (7) alcohol use (none to moderate drinking, problem drinking, or heavy drinking). Regression diagnostic procedures yielded no evidence of multicollinearity or overly influential outliers in any of the models.

## Results

### Participant Characteristics

In Botswana, 1,433 individuals in total were randomly selected. Of these, 178 people (12.4%) were excluded from the study sample: 46 (3.2%) were unavailable after two repeat visits, 78 (5.4%) refused or did not meet criteria, and 54 (3.8%) had missing data on our key predictor or outcomes of interest. In Swaziland, 876 individuals were randomly selected, and 80 (9.1%) were excluded from the sample: 35 (4.0%) were unavailable after two repeat visits, 32 (3.7%) refused or did not meet criteria, and 13 (1.5%) had missing data on our key predictor or outcomes. Demographic and behavioral characteristics of the 1,050 women and 999 men that met inclusion criteria from both countries are shown in Table 1. Thirty-two percent of women (Botswana 28%, Swaziland 38%) and 22% of men (Botswana 18%, Swaziland 29%) reported not having enough food to eat over the previous 12 mo. Eight percent of women (Botswana 11%, Swaziland 2%) and 11% of men (Botswana 10%, Swaziland 13%) reported unprotected sexual intercourse with a nonprimary partner over the previous month. In addition, 5% of women (Botswana 7%, Swaziland 2%) reported exchanging sex for money or resources, and 10% of men (Botswana 13%, Swaziland 4%) reported paying for sex with money or resources over the previous year. Finally, 17% of women (Botswana 19%, Swaziland 14%) and 15% of men (Botswana 15%, Swaziland 16%) reported intergenerational relationships; 5% of women reported having experienced forced sex (Botswana 6%, Swaziland 4%) in the past 12 months; and 26% of women (Botswana 25%, Swaziland 29%) reported that they lacked control in sexual relationships over the previous 12 mo.

**Table 1:**
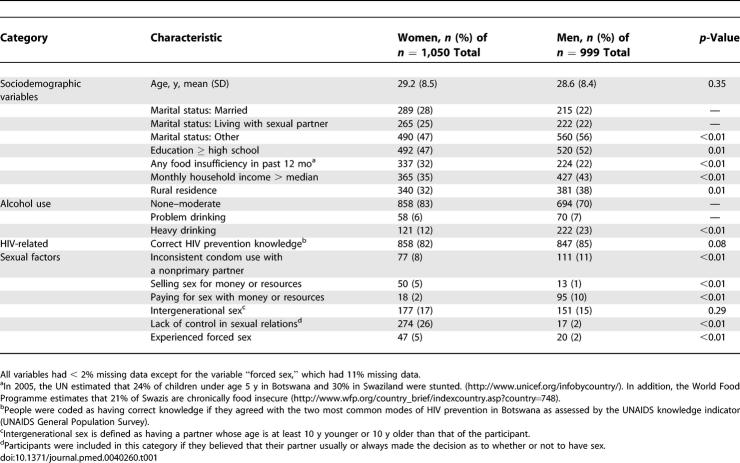
Baseline Characteristics of Respondents, Botswana and Swaziland 2004–2005

### Correlates of Risky Sexual Practices among Women in Botswana and Swaziland

Tables 2 and 3 represent the results of bivariate and adjusted analyses for women in both Botswana and Swaziland. We found a similar pattern of results in analyses stratified by country and thus are reporting the combined results. Among women, food insufficiency was associated with approximately 70% higher odds of inconsistent condom use with a nonprimary partner after adjusting for other covariates in a multivariate model (adjusted odds ratio [AOR] 1.73, 95% confidence interval [CI] 1.27–2.36) (Table 2). There was a dose–response relationship between alcohol and risky sexual practices, in that individuals who were problem drinkers had over four times the odds of reporting inconsistent condom use, and individuals who were heavy drinkers had nearly seven times the odds of reporting inconsistent condom use compared to nondrinkers or moderate drinkers. Older age, higher education, residence in a rural area, and having correct knowledge of HIV prevention were all associated with significantly lower odds of reporting unprotected sex among women.

**Table 2:**
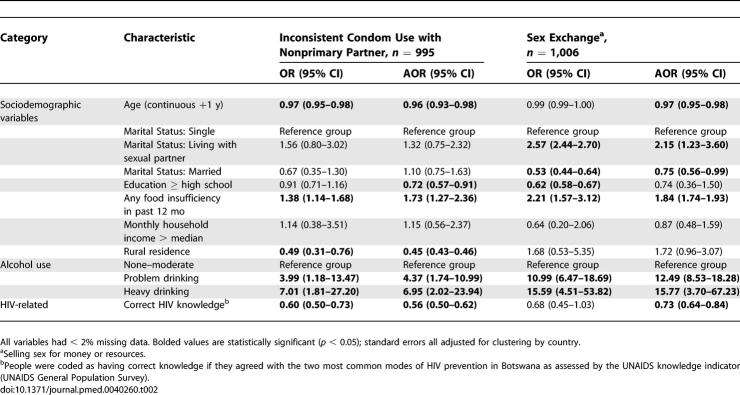
Unadjusted and Adjusted Associations with Unprotected Sex and Sex Exchange for Women, Botswana and Swaziland 2004–2005

**Table 3:**
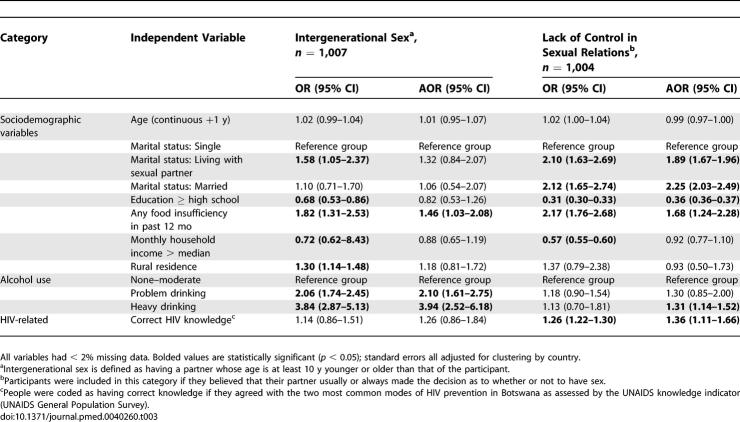
Unadjusted and Adjusted Associations with Intergenerational Sex and Lack of Control in Sexual Relations for Women, Botswana and Swaziland 2004–2005

Unadjusted and adjusted correlates of exchanging sex for money or resources among women are also displayed in Table 2. In unadjusted analyses, food insufficiency was associated with over two times the odds of engaging in sex exchange (odds ratio [OR] 2.21, 95% CI 1.57–3.12). When adjusting for all covariates, food insufficiency was still associated with nearly two times the odds of sex exchange (AOR 1.84, 95% CI 1.74–1.93). A dose–response relationship between alcohol use and sex exchange was again seen: problem drinkers had over 12 times the odds of reporting sex exchange, and heavy drinkers had over 15 times the odds of reporting sex exchange, compared to nondrinkers or moderate drinkers. As with inconsistent condom use, correct HIV knowledge was associated with significantly lower odds of reporting sex exchange. Higher education was associated with lower odds of sex exchange in unadjusted analyses only. Individuals living with sexual partners had over two times the odds, and individuals who were married had 25% lower odds of reporting sex exchange.

Food insufficiency was also significantly associated with reporting intergenerational sexual relationships among women (Table 3). Women who reported food insufficiency had over 80% higher odds of reporting intergenerational sex in unadjusted analyses, and nearly 50% higher odds of reporting intergenerational sex in adjusted analyses (AOR 1.46, 95% CI 1.03–2.08). The only additional correlates of intergenerational sex in adjusted analyses were problem drinking and heavy drinking (which again show a dose–response relationship between alcohol use and reporting intergenerational sexual relationships). Higher income and education were significantly associated with intergenerational sex in unadjusted analyses only.

Table 3 also shows unadjusted and adjusted correlates of lack of control in sexual relations among women. Women who reported food insufficiency had over two times the odds of reporting lack of control in sexual relationships in unadjusted analyses, and 70% higher odds of reporting lack of sexual control in adjusted analyses (AOR 1.68, 95% CI 1.24–2.28). Higher education was associated with lower odds of lack of sexual control in unadjusted and adjusted analyses, and higher income was associated with lower odds of lack of sexual control in unadjusted analyses only. Additional correlates of reporting lack of control in sexual relationships included being married, living with a sexual partner, having correct HIV knowledge, and heavy drinking.

While food insufficiency was associated with two times the odds of reporting forced sex, this association was not statistically significant in adjusted analyses (AOR 1.98, 95% CI 0.57–6.91). The only significant correlate of reporting forced sex among women in adjusted analyses was heavy drinking (AOR 3.58, 95% CI 1.7–7.4).

### Correlates of Risky Sexual Practices among Men in Botswana and Swaziland

Among men, the associations between food insufficiency and risky sexual practices were weak compared to women (Table 4). Food insufficiency was associated with 14% higher odds of inconsistent condom use with a nonprimary partner (95% CI 1.10–1.18), and was not associated with paying for sex with money or other resources, or with intergenerational relationships. Similar to women, alcohol use was associated with higher odds of all risky sexual outcomes examined, and a dose response relationship was observed. Individuals with higher income had lower odds of reporting inconsistent condom use, and higher odds of paying for sex with money or other resources. In contrast to women, higher education and correct HIV prevention knowledge were not associated with any of the risky sexual practices examined. In addition, older age was associated with higher odds of risky sexual practices (instead of lower odds as was seen with women), although this association was only statistically significant for intergenerational sex.

**Table 4:**
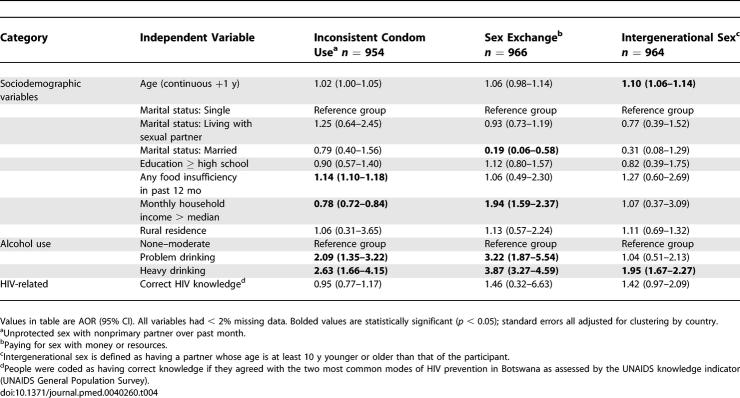
Adjusted Associations with Unprotected Sex, Sex Exchange, and Intergenerational Sex for Men, Botswana and Swaziland 2004–2005

## Discussion

While many have hypothesized a strong association between food insufficiency and HIV transmission behaviors [1,8,17], there has been little previous empirical research to substantiate these associations. Our population-based study found that food insufficiency was associated with multiple risky sexual practices for women in Botswana and Swaziland. Women who reported lacking sufficient food to eat had an 80% increased odds of selling sex for money or resources, a 70% increased odds of engaging in unprotected sex and reporting lack of sexual control, and a 50% increased odds of intergenerational sex. Our results extend previous findings by Dunkle et al. that women who reported hunger in the household were more likely to engage in transactional sex [21], and by Oyefere at al. who found that low socioeconomic status and food insufficiency played a strong role in influencing women to become sex workers [19]. Oyefere et al. also found that poverty and food insufficiency significantly influenced the decision of whether to use condoms among female sex workers [19].

Multiple prior studies have shown strong correlations among unprotected sex, intergenerational sex, forced sex, lack of sexual control, and sex exchange in sub-Saharan Africa. For instance, African women who had experienced sexual violence and who lacked control over sexual decision-making were more likely to engage in unprotected sex, to exchange sex for money or resources, to have multiple partners, and to be HIV positive [32,35–38]. Women in intergenerational relationships were also more likely to engage in unprotected sex, sex exchange, to have multiple partners, and to have experienced forced sex [32,39]. Consequently, the impact of food insufficiency on each of these sexual behaviors is likely compounded by the interdependence of these risky behaviors on one another, risky behaviors that are in turn influenced by the social and economic inequality many African women face in a context of inadequate protection of their human rights.

Given that low socioeconomic status has been shown to be associated with inconsistent condom use, sex exchange, and forced sex in studies in sub-Saharan Africa [1,21,22], we sought to better understand whether the impact of food insufficiency on risky sex could be explained by income or other measures of socioeconomic status. We found that income and education did not significantly influence the relationship between food insufficiency and risky sexual behaviors. As has been documented elsewhere [40], this result may suggest that while food insufficiency is undoubtedly influenced by socioeconomic status, it is a distinct entity with unique causes and consequences. A possible explanation for this is that in sub-Saharan Africa there is often heterogeneity of food insecurity within socioeconomic strata; many indigent individuals are able to grow food on their own, and certain family members may be preferentially fed over others. For example, a number of studies have shown that women within households in various parts of sub-Saharan Africa may be less food secure than men as a result of unequal household food allocation, a situation exacerbated by their lack of control over decisions related to food production, consumption, and sale. Men are also often served both higher quantity as well as quality of food [40–42]. Since household income may not actually capture access to money and economic dependency within the household, another possible interpretation of these findings is that food insecurity is a better marker of poverty than income and education, and it is primarily poverty that drives increases in risky sexual behaviors. Part of the difficulty in teasing out the independent effects of food insecurity from low socioeconomic status is that these categories are complex and highly correlated. Consequently, we cannot exclude the possibility that socioeconomic status plays a more significant role in mediating the relationship between food insufficiency and risky sexual practices than our findings would suggest. Nevertheless, whether food insecurity affects HIV transmission behaviors above and beyond the effects attributable to low socioeconomic status, or whether food insecurity is simply a strong marker of socioeconomic status, these findings suggest that intervening at the level of food insecurity could potentially play an important role in curtailing the spread of HIV/AIDS.

While food insufficiency was a consistent correlate of sexual risk-taking for women, for men food insufficiency was associated with only a small increase in the odds of reporting unprotected sex (14%), and was not associated with other risky sexual outcomes. Women were also significantly more likely to report food insufficiency than men in both Botswana and Swaziland. These findings highlight the strong interplay between gender inequality, food insufficiency, and sexual risk-taking in sub-Saharan Africa. By customary law and traditional norms and practices, women often find themselves subordinated to male heads of household [43], which contributes to both food insufficiency among women and risky sexual behaviors as a means to procure food [1]. The fact that women are more likely to be malnourished further heightens their risk of acquiring HIV in view of the effects of malnutrition on the immune system [1,12]. As others have argued, there is a strong need to target gender-discrimination and gender-based violence in HIV prevention programs [18,26,36,37,43], an effort that will require social, legal, structural, and cultural changes at many levels in society. For men in sub-Saharan Africa, one possible mechanism through which food insufficiency may lead to increase sexual risk-taking is that lack of food often leads to migration for work [8,44], and migration has been found to be associated with increased sexual risk-taking and HIV prevalence in multiple studies [45–49]. Our study design was not structured to adequately capture this specific causal pathway among men, and more studies are needed that examine associations among food insufficiency, labor migration, and sexual risk-taking in men.

Most HIV prevention interventions to date focus on changing people's knowledge, attitudes, and behaviors, which are believed to be the proximate causes of high HIV transmission [50]. Few interventions actually target the underlying circumstances and fundamental causes that may foster these behaviors and attitudes, and there has been a recent call to broaden the scope of interventions to consider community- and national-level factors [51]. Blankenship et al. argue for the implementation of structural interventions for HIV prevention, in that these interventions “promote health by altering the structural context in which health is produced and reproduced” [50]. They point to evidence that micro-credit programs for women and other economic and educational interventions help improve women's household bargaining power and decrease their dependence on male partners, which in turn can provide them with a basis upon which to demand safer sexual practices. Consistent with this argument, we found that higher education was associated with lower odds of risky sexual behaviors for women but not men, suggesting that education may help improve gender power imbalances and women's ability to negotiate safer sex. Attesting to the importance of structural interventions targeted at women, the IMAGE (Intervention with Microfinance for AIDS and Gender Equity) study reported that a poverty-focused microfinance initiative combined with a gender and HIV training curriculum led to a 55% reduction in levels of intimate-partner violence [52]. In addition to income generation programs and educational initiatives, our findings suggest that interventions that use targeted food supplementation and food production strategies could help address some of the gender and economic disparities that drive unsafe sexual behaviors, and should be considered as a way to reduce HIV transmission behaviors in specific high-risk populations. Any such interventions would certainly need to address equity of food distribution within the household as well as long-term sustainability.

Consistent with previous research [32,53–55]. we found that problem drinking and heavy drinking were strongly and consistently associated with all risky sexual behaviors examined for both men and women, and that a dose–response relationship was seen between alcohol use and sexual risk-taking. We have reported that alcohol use was the strongest predictor of inconsistent condom use, sex exchange, and multiple partnerships among both men and women in Botswana [32]. This study advances those previous findings by showing that the relationship between alcohol use and risky sex also holds in Swaziland, and also by linking alcohol use to having experienced forced sex among women.

In addition to the limitations in our measures of socioeconomic status discussed above, there are several additional limitations to this study. First, the causal direction of the associations between food insufficiency and risky sexual practices cannot be inferred from the cross-sectional study design. Second, social desirability bias may have influenced reporting of risky sexual behaviors and other personal information. Of note, the WHO multi-country study on women's health and domestic violence found a higher prevalence of forced sex in three countries in sub-Saharan Africa compared to our study [56]. Under-reporting of forced sex and other risky sexual behaviors may have undermined the validity of our findings [57]. Yet, even if risky sexual practices were under-reported, this would not necessarily affect the associations we found between food insufficiency and risky sex. Third, our 12-mo interval may be vulnerable to recall bias and could not detect more precise temporal associations between short-term food insufficiency and HIV transmission risk behaviors, or seasonal variability with respect to food insufficiency. These limitations, however, would bias the study toward the null hypothesis. As such, the true associations may be stronger than our data indicate. Finally, while our single question to measure food insufficiency was previously validated, it may have underestimated the prevalence of poor access to food. Food insufficiency is only one component of the broader concept of food insecurity, which also encompasses insufficient quality and diversity of foods, feelings of deprivation or restricted choice about the amount and types of food available, and inability to procure food in a socially acceptable manner [58,59]. More detailed studies using more extensive and sensitive food security scales validated in the African context with attention to possible regional and cultural differences will be important to confirm and expand upon these preliminary relationships.

### Concluding Remarks

Food insufficiency is associated with multiple high-risk sexual behaviors among women in Botswana and Swaziland. Our findings suggest that protecting and promoting access to food may decrease vulnerability to HIV, especially among women. Thus, targeted food assistance and support for women's subsistence farming and other means of food production should be considered as strategies to decrease transmission risk for women in sub-Saharan Africa. Such programs need to be fundamental parts of broader initiatives to decrease poverty among women and improve women's social and economic rights in sub-Saharan Africa.

## Abbreviations

AOR: adjusted odds ratio
CI: confidence interval
OR: odds ratio
